# Supplementary figures and images for: Targeted IS-element sequencing uncovers transposition dynamics during selective pressure in enterococci
Source: PLoS Pathog. 2023 Jun 2;19(6):e1011424. doi: 10.1371/journal.ppat.1011424 (PMC10266640; doi:10.1371/journal.ppat.1011424)

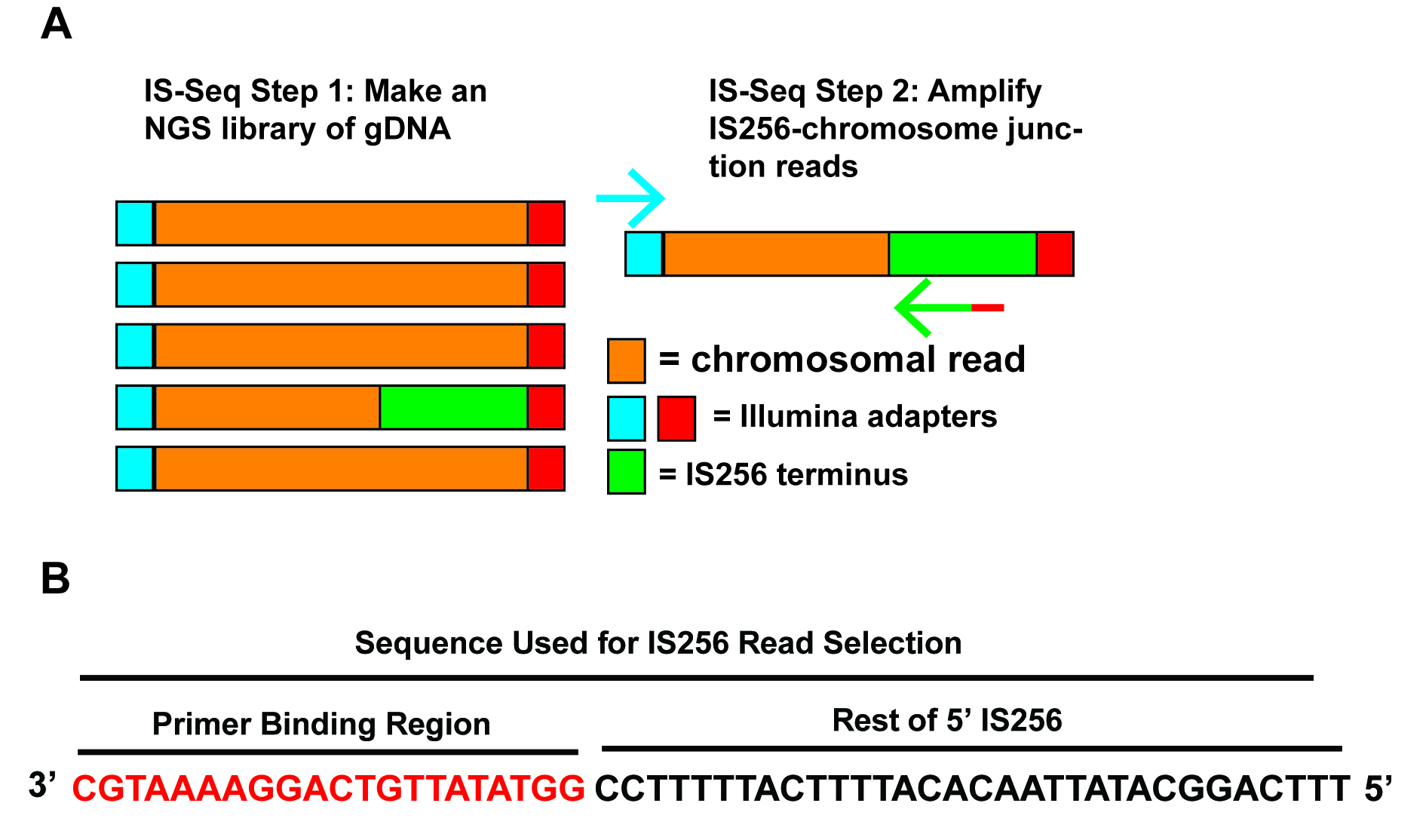

Supplement: S1 Fig — A) Schematic of the IS-Seq PCR amplification step. B) Trimming site and binning strategy used to identify IS256-termini reads. (TIF) [file ppat.1011424.s001.tif]

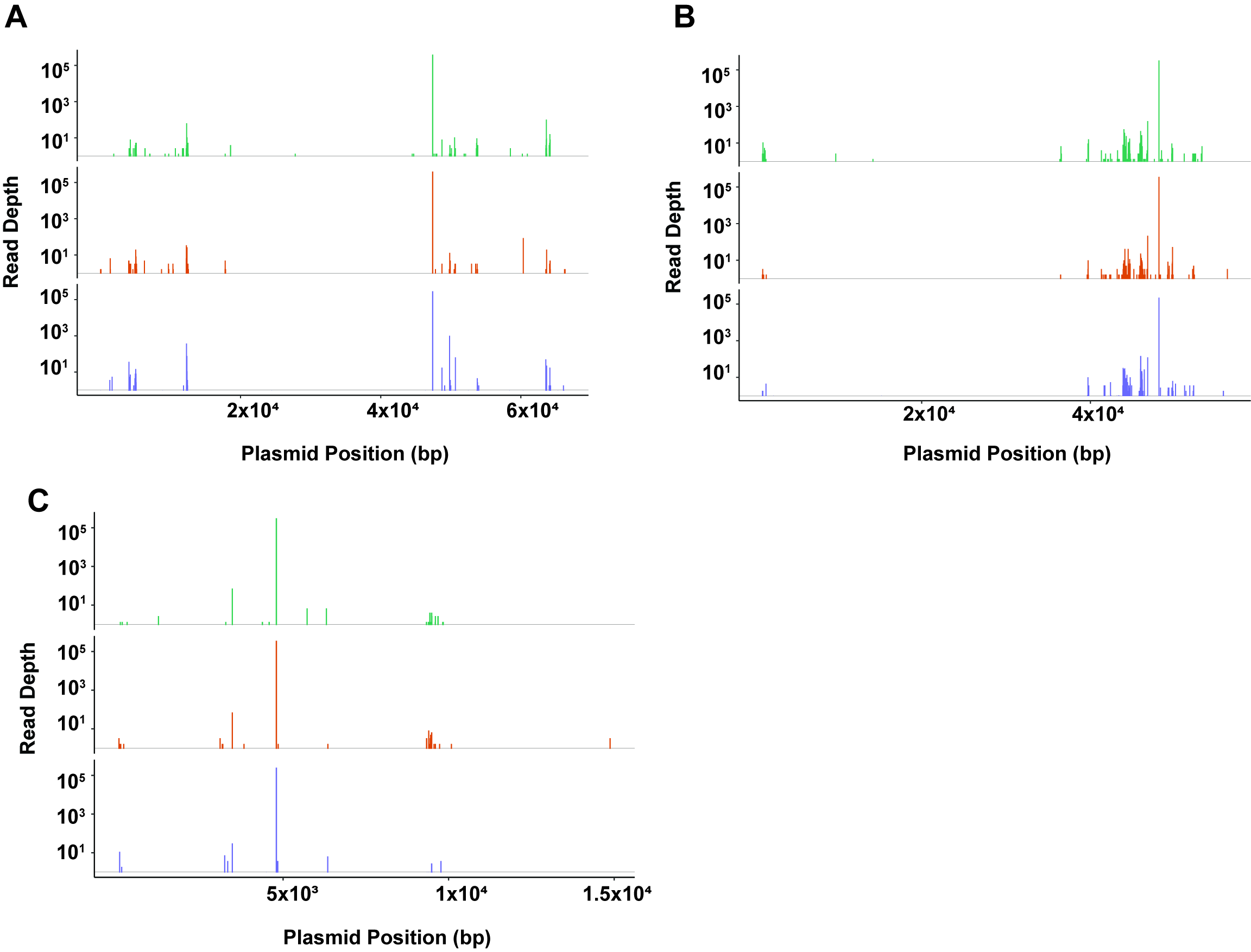

Supplement: S2 Fig — A) pTEF1, B) pTEF2, and C) pTEF3. Three individual biological replicates are shown in each panel. (TIF) [file ppat.1011424.s002.tif]

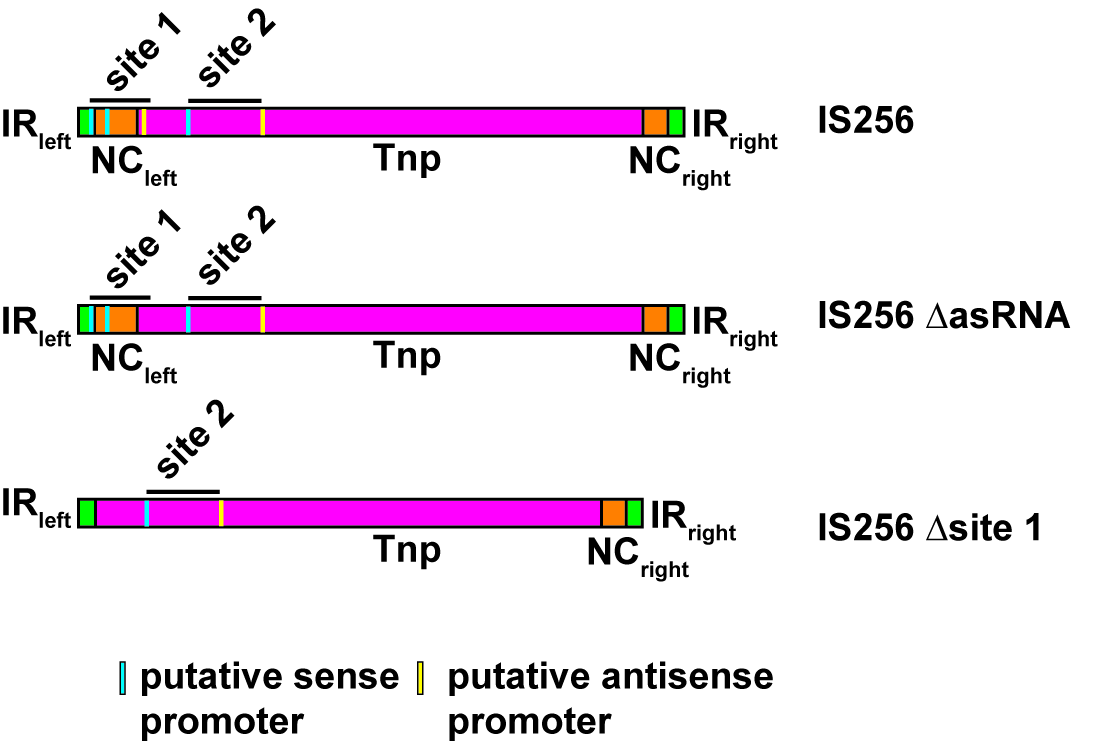

Supplement: S3 Fig — (TIF) [file ppat.1011424.s003.tif]

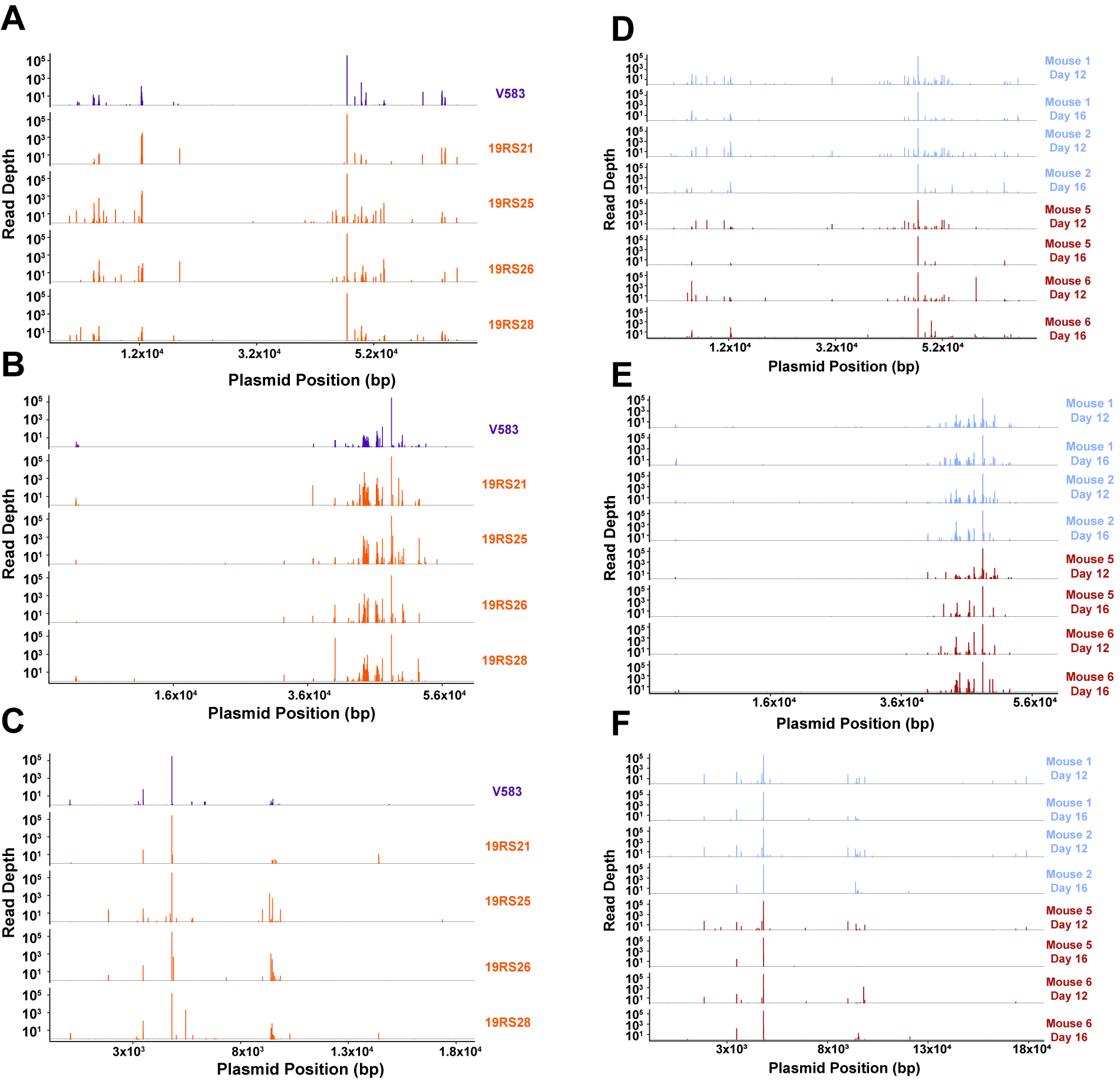

Supplement: S4 Fig — A-C) Insertion sties from in vitro cultured E. faecalis 19RS strains; A) pTEF1, B) pTEF2, and C) pTEF3. D-F) Insertion sites from E. faecalis isolated from the murine intestine D) pTEF1, E) pTEF2, and F) pTEF3. Each peak in panels A, B, and C are the average of three biological replicates per strain, and in panels D, E, and F are an average of two biological replicates. (TIF) [file ppat.1011424.s004.tif]

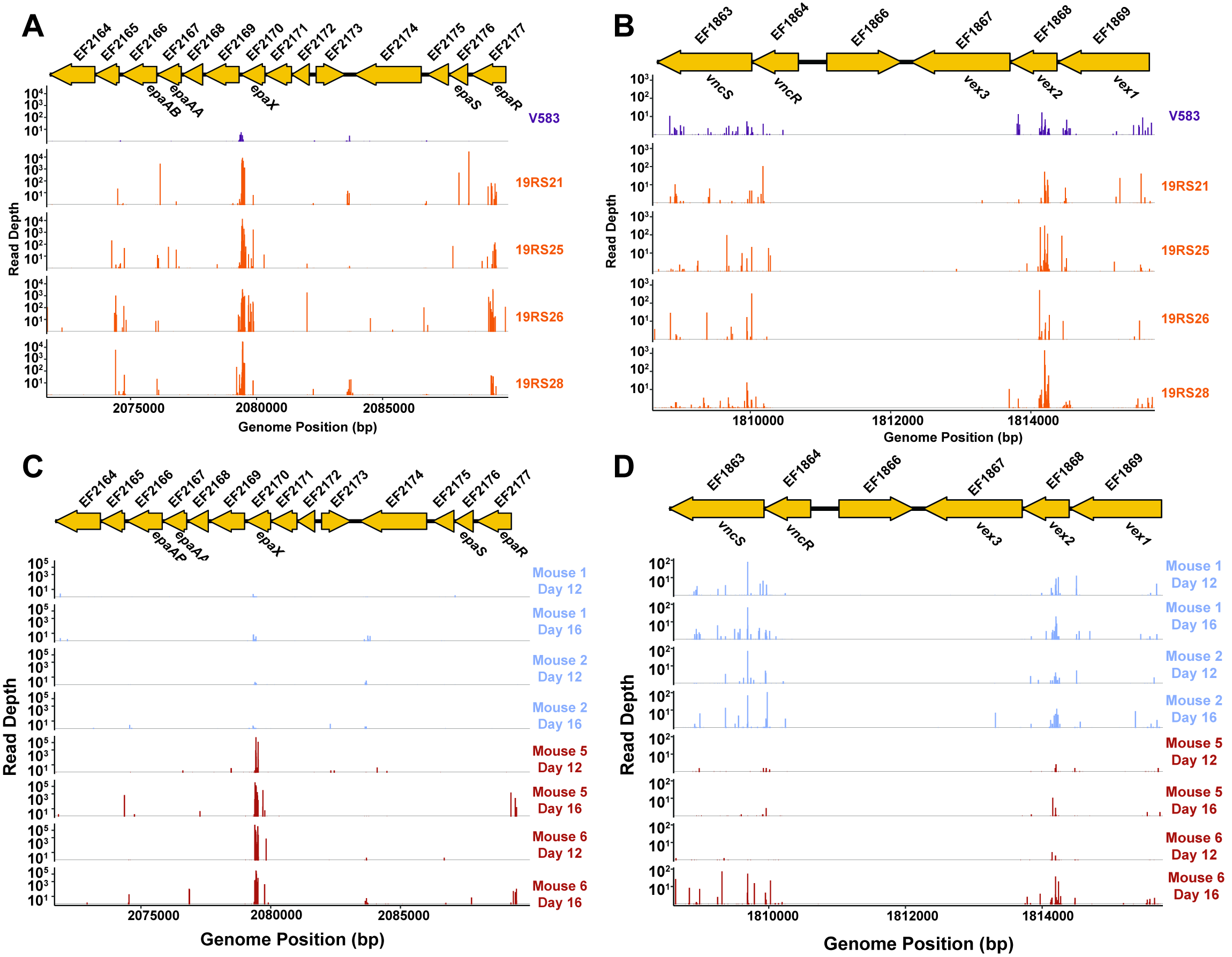

Supplement: S5 Fig — Insertion sties from in vitro cultured E. faecalis 19RS strains in the A) epa locus and B) vex/vnc locus. Insertion sites from E. faecalis isolated from the murine intestine in the C) epa locus and D) vex/vnc locus. Each peak in panels A and B are the average of three biological replicates per strain, and in panels C and D are an average of two biological replicates. (TIF) [file ppat.1011424.s005.tif]

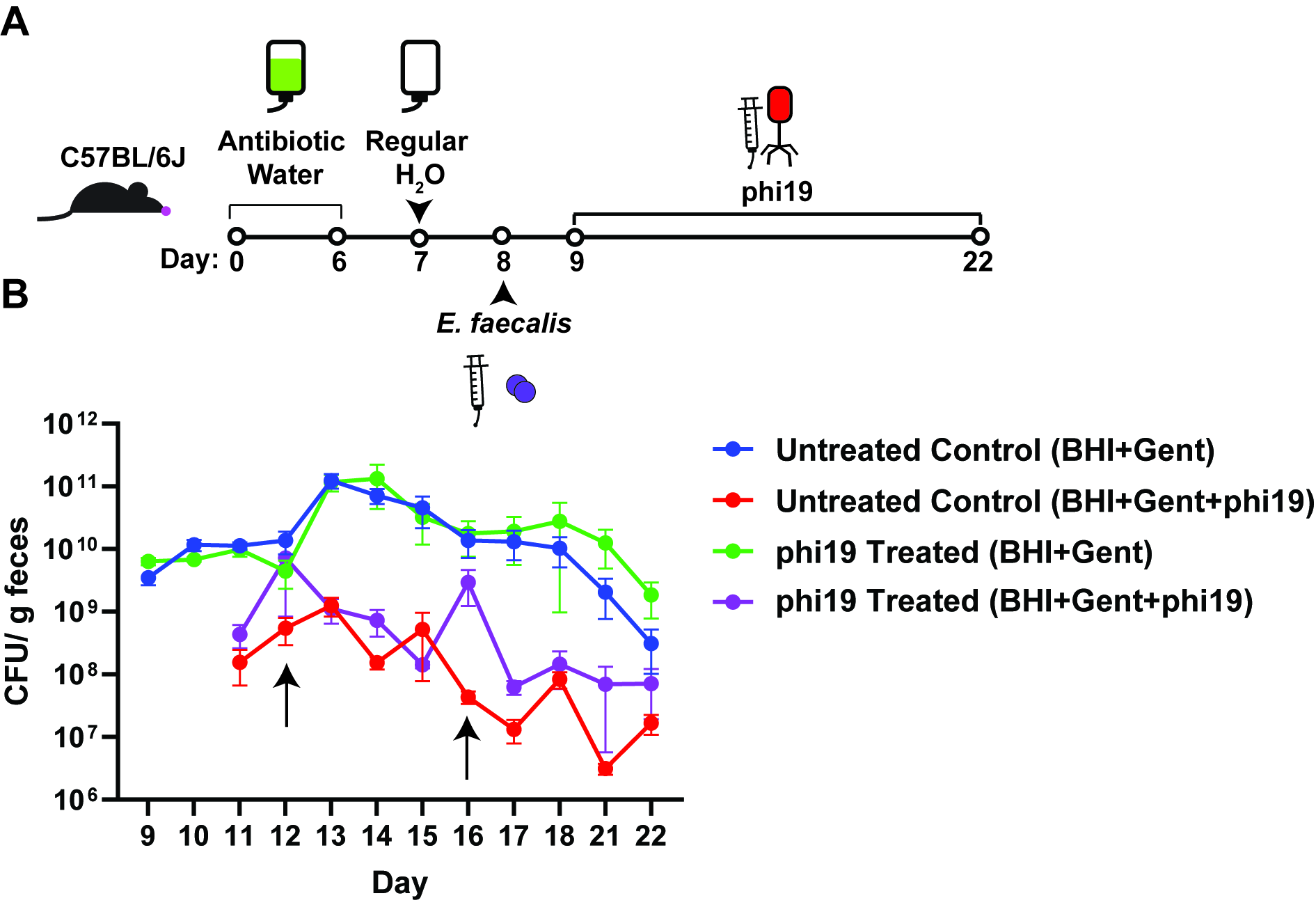

Supplement: S6 Fig — A) Schematic of the E. faecalis intestinal colonization model used to identify in vivo IS256 mobilization. The bacterial population was isolated during the phage treatment phase (between Day 9–22) by plating on selective media and genomic DNA was isolated from these cells. B) Colony forming units of E. faecalis isolated from mouse feces. Bacteria were enumerated on growth media supplemented with gentamicin with and without the addition of phi19 to measure the frequency of phage resistant colonies. Arrows indicate time points where IS-Seq was performed. Four mice were used in both the phi19 treated and untreated groups. Bacteria used for IS-Seq characterization were isolated on BHI gentamicin media. (TIF) [file ppat.1011424.s006.tif]

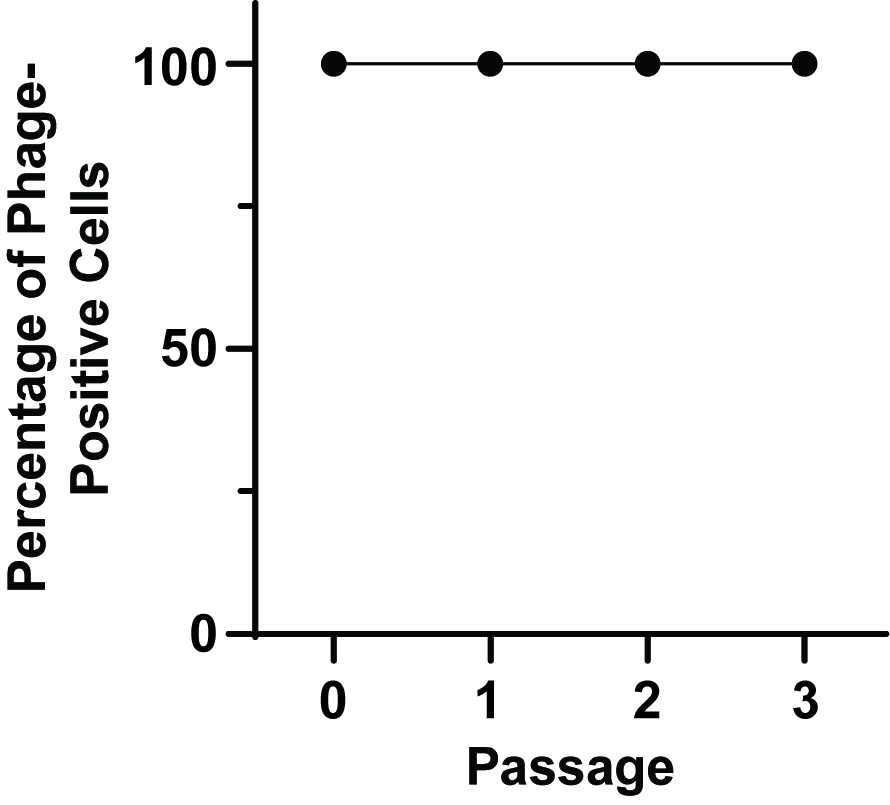

Supplement: S7 Fig — (TIF) [file ppat.1011424.s007.tif]

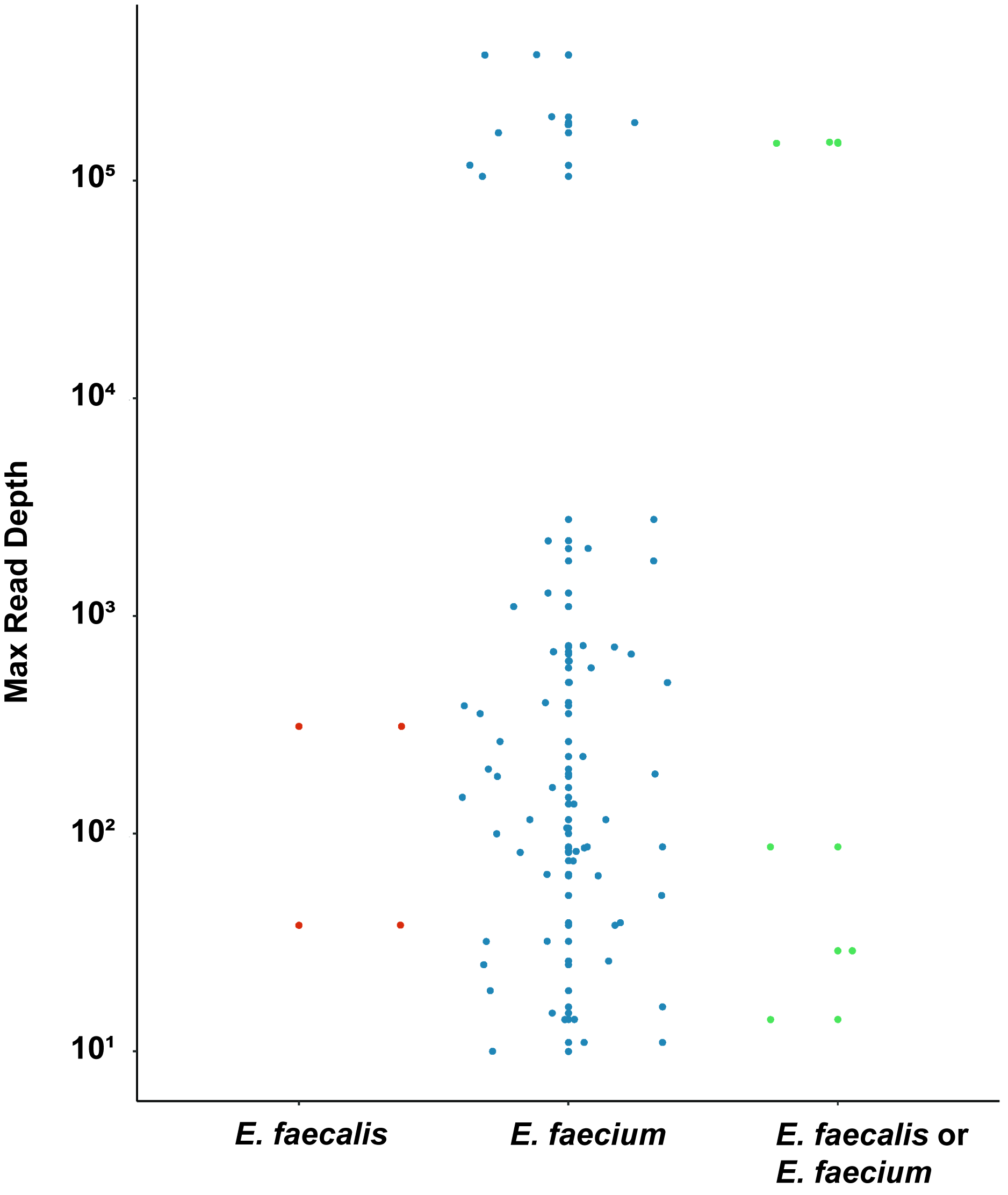

Supplement: S8 Fig — Each data point indicates the read mapping abundance per assembled contig. Data points indicated as E. faecalis or E. faecium lacked sufficient data to definitively categorize these contigs as either E. faecalis or E. faecium. (TIF) [file ppat.1011424.s008.tif]

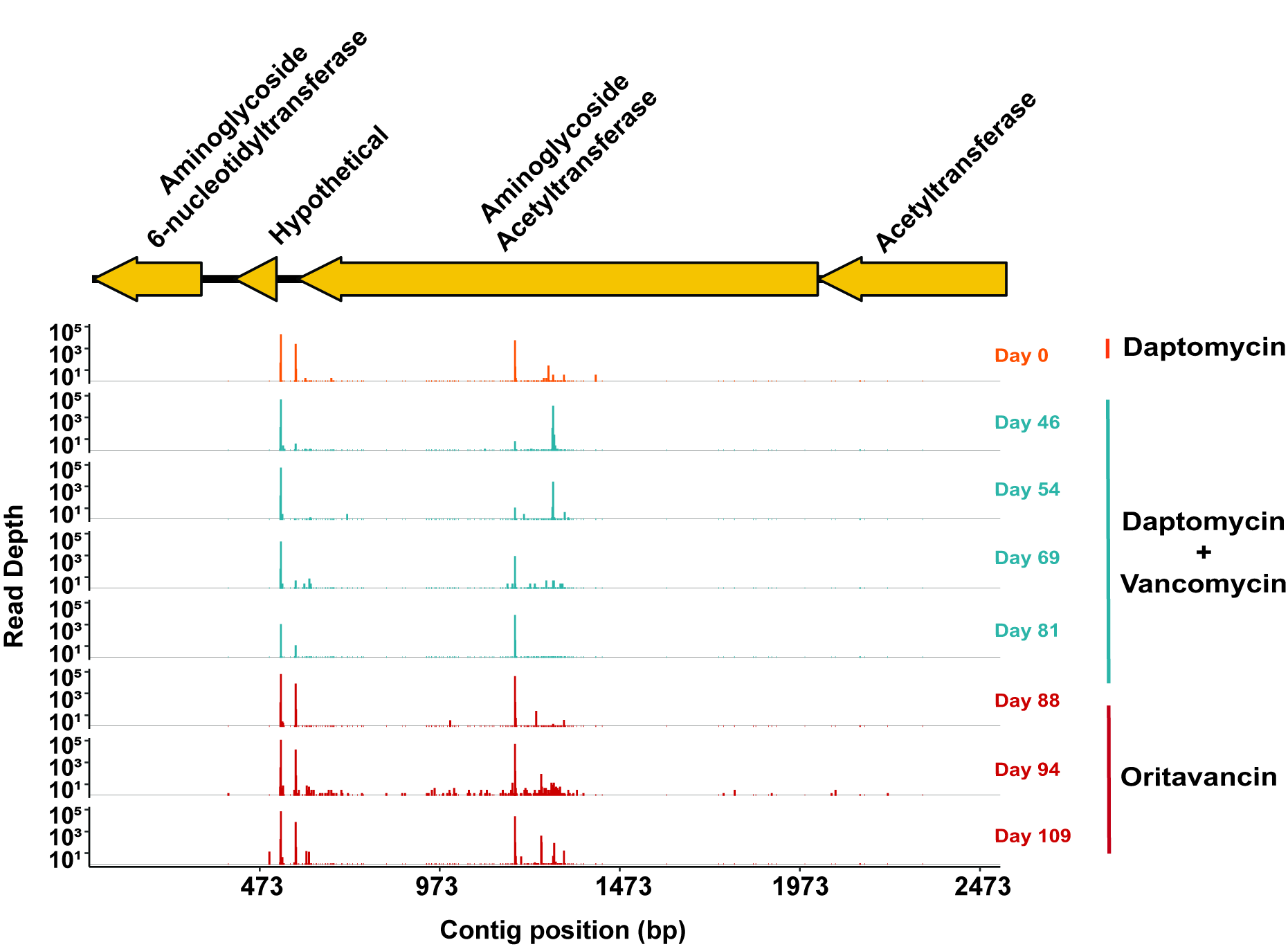

Supplement: S9 Fig — Each insertion is derived from one biological replicate per time point from the patient’s stool. (TIF) [file ppat.1011424.s009.tif]

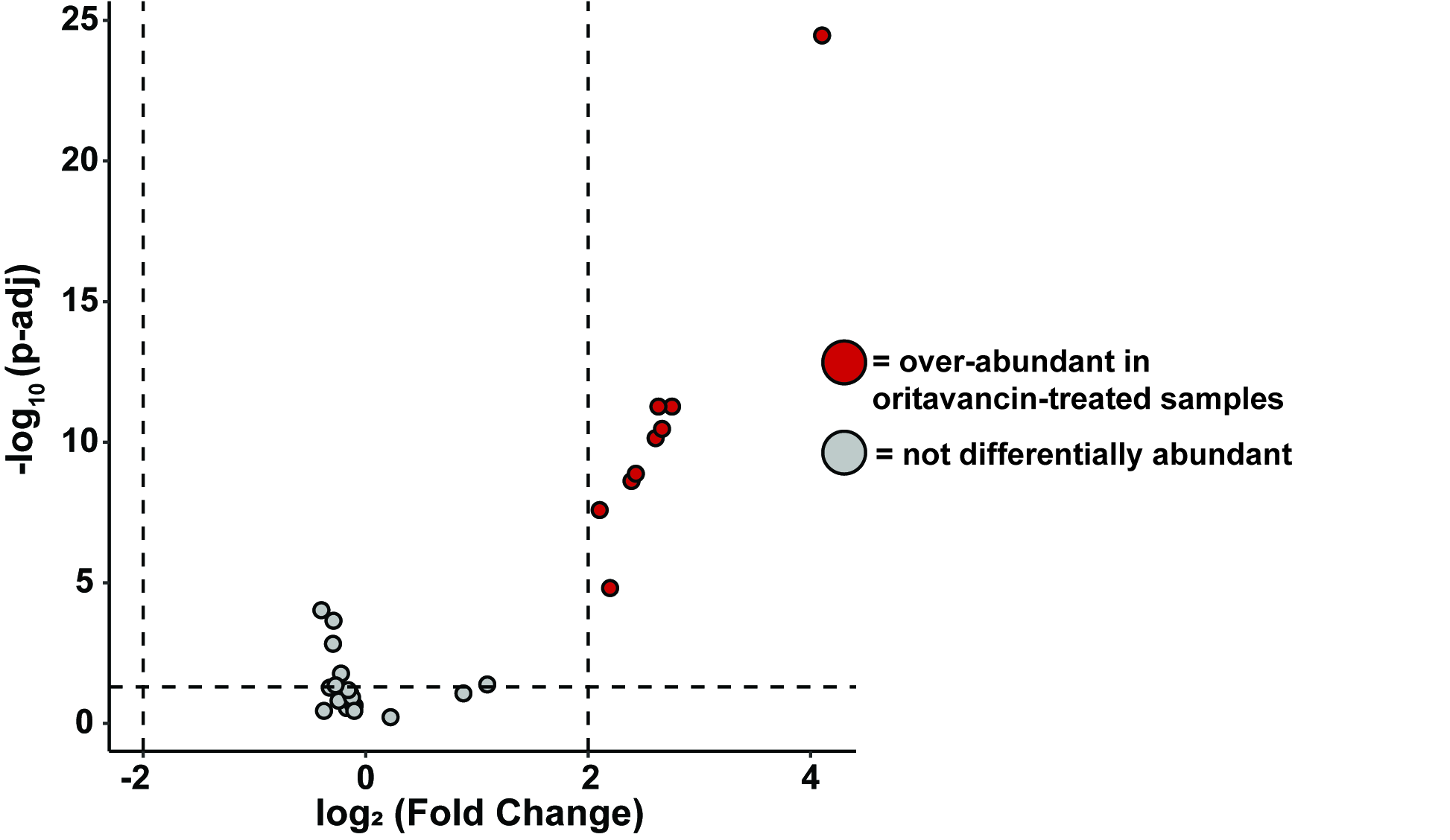

Supplement: S10 Fig — (TIF) [file ppat.1011424.s010.tif]
